# Supplementary material for: SyNDI: synchronous network data integration framework
Source: BMC Bioinformatics. 2018 Nov 6;19:403. doi: 10.1186/s12859-018-2426-5 (PMC6219086; doi:10.1186/s12859-018-2426-5)
Supplement: Supplementary file 5 — Files of the example presented in Additional file 4. This additional file is a zip package that contains all files used in Additional file 4. Detailed descriptions of each of these files are presented in a README file included in this zip package. (GZ 30411 kb) [file 12859_2018_2426_MOESM5_ESM.gz › SyNDITools/Meme2Fimo/meme_4.9.0_4.tar.gz!/meme_4.9.0/website/html/help_sequences.html]

MEME Suite - Motif Sequences 


|  |  |
| --- | --- |
|  | MEME Suite The MEME Suite web application requires the use of JavaScript   Javascript doesn't seem to be available on your browser.  - **sequences**    This is the group of sequences (the "training set") which you want   to analyze for patterns that are shared among the sequences and/or   repeat within individual sequences. All of the sequences must be   either protein or DNA. You may not mix different types of sequences   in the same group. A large number of sequence **formats** are supported. To specify the   sequences you wish to analyze, you can enter either a file name or   the actual sequences. Do not enter both.   - **file name**      This should be the name of a file on your     computer containing a group of related sequences in an appropriate     sequence **format**.   - **actual sequences**      You may type or cut-and-paste the     sequences in an appropriate sequence **format** in the window provided. |
